# Electronic cigarettes and cardiovascular diseases: An updated systematic review and network meta-analysis

**DOI:** 10.18332/tid/208065

**Published:** 2025-09-05

**Authors:** Amarit Tansawet, Thunyarat Anothaisintawee, Suparee W. Boonmanunt, Prapaporn Pornsuriyasak, Kanokporn Sukhato, Natasha Chawala, Patcharanat Inpithuk, Chatuthanai Savigamin, Saharat Liampeng, John Attia, Gareth J. McKay, Ammarin Thakkinstian

**Affiliations:** 1 Department of Research and Medical Innovation, Faculty of Medicine Vajira Hospital, Navamindradhiraj University, Bangkok, Thailand; 2 Department of Clinical Epidemiology and Biostatistics, Faculty of Medicine Ramathibodi Hospital, Mahidol University, Bangkok, Thailand; 3 Department of Family Medicine, Faculty of Medicine Ramathibodi Hospital, Mahidol University, Bangkok, Thailand; 4 Johns Hopkins Bloomberg School of Public Health, Johns Hopkins University, Baltimore, United States; 5 Centre for Clinical Epidemiology and Biostatistics, School of Medicine and Public Health, The University of Newcastle, Australia, Newcastle, Australia; 6 Hunter Medical Research Institute, The University of Newcastle, Australia, Newcastle, Australia; 7 Centre for Public Health, School of Medicine, Dentistry, and Biomedical Sciences, Queen’s University Belfast, Belfast, United Kingdom

**Keywords:** electronic cigarette, cardiovascular disease, myocardial infarction, stroke, network meta-analysis

## Abstract

**INTRODUCTION:**

The association between electronic cigarettes (e-cigarettes) and the risk of cardiovascular disease (CVD) remains inconclusive. This study aims to compare CVD risk from the use of e-cigarettes, cigarettes, combined cigarette and e-cigarette use, and non-use.

**METHODS:**

This study is a systematic review and network meta-analysis (NMA). MEDLINE and Scopus databases (through February 2024) were used to identify eligible studies. Observational studies that investigated the effect of e-cigarettes on the risk of composite CVD, myocardial infarction (MI), or stroke, compared to cigarette, dual use, or non-use, were included. NMA was applied to estimate relative effects (i.e. adjusted odds ratio, AOR) of e-cigarette, cigarette, and dual use, on composite CVD, MI, and stroke outcomes. Risk of bias was assessed using the Joanna Briggs Institute tool for surveys and the Newcastle-Ottawa scale for cohort studies.

**RESULTS:**

Eleven adult population studies were eligible for review. E-cigarette, cigarette, and dual use were significantly associated with composite CVD outcomes. Pooled AORs (95% CI) were 1.31 (1.05–1.62) for e-cigarette, 1.57 (1.30–1.88) for cigarette, and 1.67 (1.37–2.03) for dual use. Additionally, former cigarette and former dual use significantly increased the risk of composite CVD outcomes, compared to non-use. The pooled AORs (95% CI) were 1.29 (1.05–1.59) for former cigarette, and 1.46 (1.03–2.08) for former dual use, while former e-cigarette use was not significantly associated with composite CVD endpoints. For MI and stroke outcomes, only cigarette and dual use were significantly associated with these events.

**CONCLUSIONS:**

Current e-cigarette, cigarette, and dual use were significantly associated with increased risk of composite CVD outcomes, while only cigarette and dual use significantly increased the risk of MI and stroke, compared to non-use. However, these findings were primarily based on cross-sectional data limiting the temporality of effect; additional prospective cohort studies are needed to confirm our findings.

## INTRODUCTION

Cardiovascular diseases (CVDs) are a major non-communicable health burden worldwide, causing approximately 18 million deaths annually^1^. Several well-established risk factors contribute to CVDs, including unhealthy lifestyle, metabolic syndrome, and tobacco use^1,2^. The Framingham risk score for coronary heart disease^3,4^, which includes smoking, advanced age, hypertension, diabetes, and dyslipidemia as significant prognostic factors, highlights the substantial impact of smoking on CVD development. Efforts to reduce smoking would benefit both individual patients and the broader healthcare system.

Electronic nicotine delivery systems, commonly known as e-cigarettes, deliver nicotine without the tar and toxic substances present in cigarettes^5,6^. Evidence has shown that e-cigarettes can be effective tools for smoking cessation^7^, and may reduce harmful exposures to cigarettes. However, nicotine and volatile organic compounds found in e-cigarette products may still pose health risks to e-cigarette users^8^.

Nicotine can induce the release of catecholamines, leading to elevated blood pressure and pulse rate^9^. It may also contribute to endothelial dysfunction by increasing oxidative stress^9^, which could potentially result in atherosclerotic changes and increase the risk of CVD. Although long-term data are limited, several meta-analyses^10-12^ based on preclinical and clinical surrogate endpoints have raised concerns about the CVD risk associated with e-cigarette use.

Recent meta-analyses have suggested that e-cigarettes may increase risk of myocardial infarction (MI)^13,14^ but evidence regarding stroke remains inconclusive^15,16^. However, only one meta-analysis^16^ included studies post 2021, with the remaining meta-analyses^13-15^ somewhat reliant on overlapping survey databases. Additionally, all these meta-analyses employed only pairwise comparisons, limiting their ability to estimate the effect sizes across all possible cigarette exposures. To address this limitation, we conducted an updated systematic review (SR) and network meta-analysis (NMA) to assess the impact of cigarettes on CVD outcomes. We estimated the effects of e-cigarette use compared to cigarette use, dual use, and non-use.

## METHODS

This systematic review and NMA is reported in line with the Preferred Reporting Items for Systematic Reviews and Meta-Analyses guidelines (PRISMA) 2020 (Supplementary file Materials 1)^17^. The review protocol has been registered at PROSPERO (CRD42024521271).

### Study identification

A two-step search process was employed to identify relevant studies. First, relevant SRs were identified from MEDLINE (via PubMed) and Scopus databases through 7 November 2023. We included SRs with or without meta-analyses that evaluated the effect of e-cigarettes on the risk of CVD. The original studies included in these eligible SRs, along with those identified from their reference lists, were further assessed for eligibility.

Second, an updated search was conducted in the same databases, covering studies published since the last search date of the most recent SR^14,15^, i.e. 1 January 2020 through 7 February 2024. Details of the search terms and strategies used for each database are provided in Supplementary file Tables 1 and 2.

### Study selection

Titles and abstracts of identified studies (694 records for step one and 561 records for step two) were screened by two independent reviewers (ATa and TA). The full texts of potentially eligible records (80 studies for step one and 14 studies for step two) were reviewed. Studies that met the pre-defined eligibility criteria were included. Disagreement between reviewers was resolved by third party consensus.

### Eligibility criteria

Observational studies (i.e. cross-sectional, case-control, or cohort studies) and randomized controlled trials (RCTs) were eligible for inclusion if they met the following criteria: 1) conducted in the general population; 2) compared the effect of e-cigarette use with any comparator including cigarette, dual use of e-cigarettes and cigarettes, or non-use; and 3) assessed CVD outcomes, including MI, stroke, or composite CVD endpoints. There were no language restrictions; however, non-English studies were excluded if their key information could not be adequately translated using available translation tools. For evidence synthesis, the included studies were categorized based on their reported outcomes.

### Exposures and outcomes of interest

The primary exposures of interest were e-cigarette, cigarette, and dual users, which were categorized into current and former users based on the definitions provided in each original study. The comparator was any never user of either product. Ultimately, exposures were classified into 7 groups including never user, former e-cigarette, former cigarette, former dual use, e-cigarette, cigarette, and dual users. The outcomes of interest were CVDs, including MI, stroke, and composite CVD endpoints defined according to the original studies.

### Data extraction

Two independent reviewers extracted the following data: study characteristics (i.e. first author’s names, publication year, study design, database used, and the number of participants), population characteristics (i.e. mean age, sex, mean body mass index (BMI), and comorbidity status), and type of exposures (i.e. e-cigarette, cigarette, dual use, former e-cigarette, former cigarette, former dual use, and never use).

Data for pooling (i.e. number of participants between exposure groups and outcomes) were extracted as a contingency table if available. Otherwise, a crude or adjusted summary statistic and standard error (SE) or upper and lower bound of 95% confidence interval (CI) were extracted. These included odds ratios (ORs) and hazard ratios (HRs). Covariates adjusted in the model were extracted. For HRs, they were converted to risk ratios (RRs) and subsequently ORs using the following equations^18^:


$$RR=\left[1-e^{HR.ln\left(1-r_{0}\right)}\right]/r_{0}$$

and


$$OR=\frac{RR-\left(r_{0}\times RR\right)}{1-\left(r_{0}\times RR\right)}$$

where *r_0_* is the incident rate of the outcome in the reference group.

### Risk of bias assessment

Risk of bias for the studies included was assessed by two independent reviewers using the Joanna Briggs Institute (JBI) appraisal tool^19^ for cross-sectional studies, which consists of eight questions. A study was considered to have a high risk of bias if it received at least one negative answer, while a study was classified as low risk of bias only if all questions received positive answers. The Newcastle-Ottawa Scale (NOS)^20^ was used to assess cohort studies. The NOS tool has three domains including cohort selection (4 items), comparability (1 item), and outcome assessment (3 items). For each domain, the number of rated stars indicates quality of that domain. A maximum of one star per item was rated except for the comparability domain which was allowed up to two stars. Studies were considered poor quality if they received ≤1 star for cohort selection or outcome assessment domains or zero stars for the comparability domain. Again, disagreement between both reviewers was resolved by third party consensus.

### Statistical analysis

To avoid potential confounding effects, only adjusted effect sizes were considered in the NMAs. Data pooling was performed separately for each outcome (i.e. composite CVD, MI, and stroke). A multivariate random-effects model with a consistency assumption was used to estimate the adjusted relative effects (i.e. AORs) of all pairwise comparisons among exposures. Surface Under the Cumulative Ranking (SUCRA) curves and P-scores ranked exposure effects for each outcome. Transitivity was assessed by comparing study characteristics between comparisons/studies. Global heterogeneity was assessed using *τ*^2^ and global chi-squared test. A design-by-treatment interaction model and node-splitting (back-calculation method) were used to investigate violations of the consistency assumption. Publication bias was assessed using a comparison-adjusted funnel plot and the Egger’s test. Asymmetry of the comparison-adjusted funnel plot was considered indicative of publication bias. P-values less than 0.05 were considered significant. All statistical analyses were performed using R version 4.4.1 (*netmeta* package)^21^. Confidence in evidence synthesis from NMA was assessed using the Confidence In Network Meta-Analysis (CINeMA) framework^22^.

## RESULTS

### Study selection

**Figure. 1:**
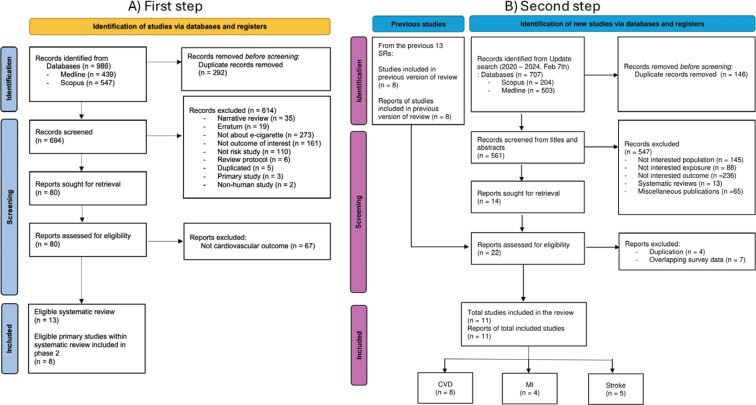
PRISMA diagram of study selection: A) first step; B) second step

The initial search (step one) identified 986 records; 292 and 614 records were excluded by deduplication and title and abstract screening, respectively. Among 80 records entering full-text screening, only 13 SRs were eligible. Eight individual studies were identified from these SRs (Figure 1A). In the second search phase (step two), 707 records were retrieved. After removing duplicates (146 records), 561 records remained for title and abstract screening. Of these, 547 records were excluded and 14 studies plus eight individual studies identified from the previous SRs, were assessed by full-text screening. Duplications and studies that used overlapping data sources were further excluded at this point. Therefore, a total of 11 primary studies^23-33^, comprising 6022304 participants, were included in this review (Figure 1B).

### Characteristics of included studies

Data were collected from six different databases, primarily based in the United States^23-26,^ ^28-33^, with the exception of one study^27^, which used data from the Korean National Health Insurance Service (Table 1). All studies focused on adult populations, with male representation ranging between 40% and 70%. Hypertension prevalence ranged from 25.2% to 39.8%; diabetes prevalence was lower, ranging from 2.1% to 20.4%. The history of e-cigarette, cigarette, dual, and never use was collected by self-report questionnaire for all studies. The questions used for defining each type of exposure for each study are presented in Supplementary file Table 3.

**Table 1:** Characteristics of included studies

*Author Year*	*Country Data source*	*Study design*	*Exposure*	*Total* *n*	Age* (years)	*Male* *%*	BMI*	*DM* *%*	*HT* *%*	*DLP* *%*	*Reported outcome*	*Outcome verification*
Wang et al.^23^ 2018	Worldwide (majority: US) Health eHeart Study	Cross-sectional	E-cigarette	573	41.4 (18)	-	-	-	-	-	MI (crude effect) Stroke (crude effect) Composite CVD (crude effect)	Self-report
			Cigarette	1693	45 (21)	-	-	-	-	-		
			Dual use	514	46 (18)	-	-	-	-	-		
			Never use	36967	-	-	-	-	-	-		
Farsalinos et al.^24^ 2019	US National Health Interview Surveys (2016 & 2017)	Cross-sectional	E-cigarette	714	43.3 (15.7)	58.2	30.6 (14.4)	7.6	27.5	27.1	Composite CVD (crude effect)	Self-report
			Ex E-cigarette	7026	41 (15.6)	55.3	29.5 (12.1)	7.1	25.2	22.2		
			Never use	50830	52.2 (18.6)	46.9	30.4 (14.2)	9.8	31.9	28.9		
Osei et al.^25^ 2019	US Behavioral Risk Factor Surveillance System (2016 & 2017)	Cross-sectional	E-cigarette	15863	30–34	58.8	18.5–30 69.4%	7.9	-	-	Composite CVD (crude & adjusted effect)	Self-report
			Never use	433229	45–49	44.8	18.5–30 68.6%	10.4	-	-		
Parekh et al.^26^ 2020	US Behavioral Risk Factor Surveillance System (2016 & 2017)	Cross-sectional	E-cigarette	3437	25–44 31.1%	67.2	18.5–30 75.1%	2.1	-	-	Stroke (crude & adjusted effect)	Self-report
			Cigarette	13318	25–44 87.4%	57	18.5–30 65.5%	5.4	-	-		
			Dual use	7493	25–44 70.5%	62.5	18.5–30 68.7%	5.7	-	-		
			Never	133077	25–44 72%	43.7	18.5–30 71.1%	4.1				
Choi et al.^27^ 2021	Korea Korean National Health Insurance Service (2014, 2015, 2018)	Cross-sectional	E-cigarette	12833	42.2 (9.6)	-	25.5 (3.3)	-	-	-	Composite CVD (crude & adjusted effect)	ICD-10
			Cigarette	1541012	48.1 (11.4)	-	24.7 (4.1)	-	-	-		
			Dual use	445885	41 (8)	-	25.7 (3.5)	-	-	-		
			Ex Cigarette	1238318	53.9 (11.7)	-	25 (4.2)	-	-	-		
			Never use	1457602	48.4 (14.5)	-	24.8 (3.2)	-	-	-		
Critcher and Siegel^28^ 2021	US National Health Interview Surveys (2014–2019)	Cross-sectional	E-cigarette	2262	50.6 (18.5)	46	30 (6.3)	10.7	34.9	30.1	MI (crude effect)	Self-report
			Ex E-cigarette	19212								
			Never use	150573								
Berlowitz et al.^29^ 2022	US Population Assessment of Tobacco and Health (2013–2019)	Cohort	E-cigarette	822	<35 62%	49	-	-	-	-	Composite CVD (crude & adjusted effect)	Self-report
			Cigarette	6515			-	-	-	-		
			Dual use	1858	<35 54%		-	-	-	-		
			Never use	14832	<35 51%		-	-	-	-		
Falk et al.^30^ 2022	US National Health Interview Surveys (2014, 2016–2018)	Cross-sectional	E-cigarette	2619	-	-	-	-	-	-	MI (adjusted effect) Stroke (adjusted effect) Composite CVD (adjusted effect)	Self-report
			Cigarette	6459	-	-	-	-	-	-		
			Dual	6581	-	-	-	-	-	-		
			Ex Cigarette	17788	-	-	-	-	-	-		
			Never use	47937	-	-	-	-	-	-		
Liu et al.^31^ 2022	US Behavioral risk factor surveillance system (2020)	Cross-sectional	E-cigarette	253561	≤55 58.6%	49.3	-	13.5	-	-	Composite CVD (crude & adjusted effect)	Self-report
			Cigarette				-		-	-		
			Dual use				-		-	-		
			Ex E-cigarette				-		-	-		
			Ex Cigarette				-		-	-		
			Ex Dual use				-		-	-		
			Never use				-		-	-		
Patel et al.^32^ 2022	US National Health and Nutrition Examination Survey (2015–2018)	Cross-sectional	E-cigarette	7756	-	-	-	-	-	-	Stroke (crude & adjusted effect) Composite CVD (crude & adjusted effect)	Self-report
			Cigarette	48625	-	-	-	-	-	-		
			Dual use	23444	-	-	-	-	-	-		
Hirschtick et al.^33^ 2023	US Population Assessment of Tobacco and Health (2013–2019)	Cohort	E-cigarette	11076	58	45	-	16.4	36.8	-	MI (crude & adjusted effect) Stroke (crude & adjusted effect)	Self-report
			Cigarette				-	16.9	36.5	-		
			Dual use				-	19.4	36.2	-		
			Never				-	20.4	39.8	-		

* Data are given as mean (SD) or range with %. BMI: body mass index (kg/m^2^). CVD: cardiovascular disease. DLP: dyslipidemia. DM: diabetes mellitus. HT: hypertension. ICD-10: international classification of diseases – 10th revision. MI: Myocardial infarction. SD: standard deviation.

Among 11 included studies, 8 ^23-25,27,29-32^, 4 ^23,28,30,33^ and 5 studies^23,26,30,32,33^ reported outcomes as composite CVD, MI, and stroke, respectively. Definitions and methods of outcome verification for each study are described in Supplementary file Table 4. Most of the included studies verified these outcomes by self-reported questionnaire. However, provision of adjusted ORs for meta-analysis were only provided by 6 studies for composite CVD^25,27,29,30-32^, 2 studies for MI^30,33^, and 4 studies for stroke^26,30,32,33^. Details of covariate adjustment used in each study are provided in Supplementary file Table 5. Data for pooling are provided in Supplementary file Tables 10–13.

### Risk of bias assessment

Only one study was classified as having low risk of bias^27^, while nearly all studies^23-26,28-33^ (10 out of 11) were judged to have a high risk of bias or poor quality, primarily due to the domain of outcome measurement (Supplementary file Tables 6 and 7). This domain was rated as high risk of bias because the CVD outcomes were assessed through self-reported questionnaires, which may not represent a confirmed CVD diagnosis, given the limitations associated with self-reported data.

### Composite CVD endpoints

**Figure. 2:**
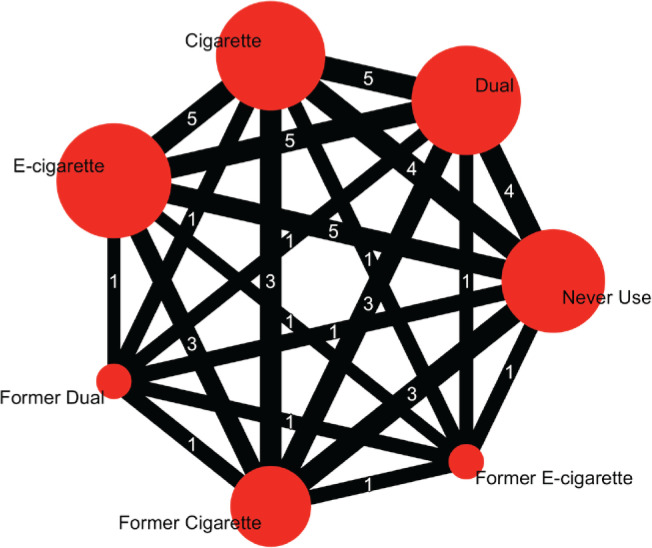
Network configuration of the composite cardiovascular outcome considering adjusted effect of exposures. The size of the nodes is proportional to the number of included studies, while the thickness of the edges corresponds to the inverse standard errors of effect sizes comparing two exposures. The numbers within the edges indicate the number of studies for each comparison

Adjusted ORs for seven exposure categories were evaluated using data from six studies^25,27,29,30-32^ (Figure 2). Common covariates in the adjusted model included age, sex, ethnicity, body mass index, education, income level, comorbidity status, and substance abuse.

**Figure. 3:**
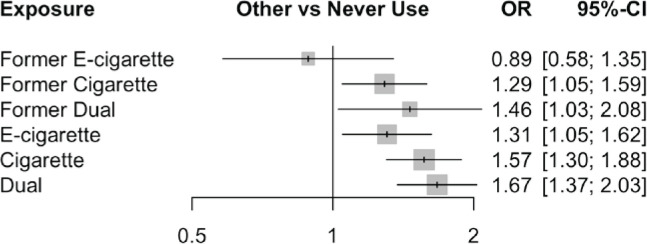
Pooled adjusted odds ratios (ORs) and 95% confidence intervals (CIs) from random-effect network meta-analysis of composite cardiovascular outcomes comparing exposures and never use. Square size around point estimates is proportional to the precision

Relative effects from seven exposures were estimated. Compared to non-use, all exposures with the exception of former e-cigarette use, were significantly associated with composite CVD endpoints. The pooled AORs (95% CI) were: 1.31 (1.05–1.62) for e-cigarette use, 1.67 (1.37–2.03) for dual use, 1.46 (1.03–2.08) for former dual use, 1.57 (1.30–1.88) for cigarette use, and 1.29 (1.05–1.59) for former cigarette use (Table 2 and Figure 3). Additionally, current dual users had a significantly higher risk of CVD compared to current e-cigarette users, former cigarette users, and former e-cigarette users, with adjusted ORs of 1.28 (1.03–1.59), 1.30 (1.04–1.61), and 1.89 (1.23–2.90), respectively, although this risk did not significantly differ for comparisons with current cigarette and former dual users. Furthermore, current cigarette use was associated with a 1.20 (0.97–1.48) times higher risk of CVD relative to current e-cigarette use, although this failed to reach the significance threshold. Among the exposures of interest, dual use was ranked as having the highest risk of CVD (SUCRA=0.91, P-score=0.91), followed by cigarette (SUCRA=0.80, P-score=0.80), former dual use (SUCRA= 0.68, P-score=0.67), e-cigarette (SUCRA=0.47, P-score=0.47) , and former cigarette smoking (SUCRA=0.45, P-score=0.45).

**Table 2:** Adjusted odds ratios for each comparison in the network meta-analysis of composite cardiovascular outcomes, myocardial infarction, and stroke. Results indicate effect size comparisons between the upper and the lower exposure along the diagonal. Direct and mixed effects are presented above and below diagonal, respectively

***Composite cardiovascular outcomes***						
**Dual use**	1.07 (0.88–1.29)	1.27 (1.01–1.59)	1.25 (0.77–2.02)	**1.36 (1.07–1.73)**	**2.06 (1.21–3.52)**	**1.75 (1.41–2.15)**
1.07 (0.88–1.29)	**Cigarette**	1.20 (0.96–1.50)	0.94 (0.62–1.43)	**1.26 (1.01–1.58)**	1.55 (0.96–2.50)	**1.64 (1.35–1.99)**
**1.28 (1.03–1.59)**	1.20 (0.97–1.48)	**E-cigarette**	0.87 (0.50–1.54)	0.87 (0.48–1.59)	0.93 (0.68–1.27)	1.44 (0.76–2.74)
1.14 (0.80–1.64)	1.07 (0.75–1.53)	0.89 (0.61–1.30)	**Former dual use**	1.07 (0.71–1.61)	**1.65 (1.01–2.69)**	1.43 (0.96–2.14)
**1.30 (1.04–1.61)**	1.21 (0.99–1.50)	1.01 (0.79–1.29)	1.13 (0.79–1.62)	**Former cigarette**	1.54 (0.97–2.46)	**1.33 (1.07–1.65)**
**1.89 (1.23–2.90)**	**1.77 (1.16–2.70)**	1.47 (0.95–2.29)	**1.65 (1.01–2.69)**	1.46 (0.95–2.23)	**Former e-cigarette**	0.87 (0.55–1.38)
**1.67 (1.37–2.03)**	**1.57 (1.30–1.88)**	**1.31 (1.05–1.62)**	**1.46 (1.03–2.08)**	**1.29 (1.05–1.59)**	0.89 (0.58–1.35)	**Never use**
***Myocardial infarction***						
**Dual use**	1.28 (0.81–2.03)	**3.78 (1.88–7.59)**	NA	**2.19 (1.34–3.58)**	NA	**3.44 (2.21–5.34)**
1.36 (0.87–2.13)	**Cigarette**	**2.94 (1.49–5.80)**	NA	1.62 (1.00–2.62)	NA	**2.45 (1.71–3.52)**
**3.85 (1.91–7.73)**	**2.83 (1.44–5.55)**	**E-cigarette**	NA	0.56 (0.27–1.17)	NA	0.91 (0.47–1.78)
NA	NA	NA	**Former dual use**	NA	NA	NA
**2.08 (1.29–3.35)**	1.53 (0.97–2.40)	0.54 (0.27–1.08)	NA	**Former cigarette**	NA	**1.75 (1.11–2.77)**
NA	NA	NA	NA	NA	**Former e-cigarette**	NA
**3.30 (2.15–5.08)**	**2.43 (1.69–3.49)**	0.86 (0.44–1.66)	NA	**1.59 (1.03–2.45)**	NA	**Never use**
***Stroke***						
**Dual use**	1.17 (0.80–1.69)	1.49 (0.97–2.28)	NA	1.86 (1.00–3.46)	NA	**2.33 (1.47–3.69)**
1.15 (0.79–1.66)	**Cigarette**	1.30 (0.86–1.97)	NA	1.64 (0.89–3.03)	NA	**1.97 (1.35–2.87)**
1.47 (0.96–2.24)	1.28 (0.85–1.92)	**E-cigarette**	NA	0.82 (0.36–1.90)	NA	0.99 (0.57–1.73)
NA	NA	NA	**Former dual use**	NA	NA	NA
1.71 (0.99–2.94)	1.49 (0.88–2.53)	1.16 (0.65–2.10)	NA	**Former cigarette**	NA	1.29 (0.71–2.33)
NA	NA	NA	NA	NA	**Former e-cigarette**	NA
**2.11 (1.41–3.15)**	**1.84 (1.29–2.63)**	1.44 (0.92–2.24)	NA	1.23 (0.72–2.11)	NA	**Never use**

NA: not available. Bold indicates significance. Results represent pooled adjusted odds ratios (95% confidence intervals) estimated from direct effects (above the diagonal) and mixed effects (below the diagonal) between each exposure comparison (read from left to right). For example, the pooled adjusted odds ratios for the composite CVD outcomes of dual use compared to never use is 1.75 (1.41–2.15) for direct effect and 1.67 (1.37–2.03) for mixed effect models.

### Myocardial infarction

For pooled adjusted ORs (Supplementary file Figure 1), e-cigarette use was not significantly associated with risk of MI (AOR=0.86; 95% CI: 0.44–1.66), in contrast to dual use, cigarette use, and former cigarette use represented by significantly increased odds of MI when compared to never use with AORs (95% CI) of 3.30 (2.15–5.08), 2.43 (1.69–3.49), and 1.59 (1.03–2.45), respectively. In addition, the odds of MI were significantly higher for dual users than those of e-cigarette, and former cigarette users with pooled AORs of 3.85 (1.91–7.73), and 2.08 (1.29–3.35), respectively. Cigarette users also had significantly higher odds of MI than e-cigarette users (Table 2). Although MI risk was greater for dual users compared to cigarette users with an AOR of 1.36 (0.87–2.13), this was not significant. Regarding SUCRA and P-scores, dual use ranked highest for MI risk (SUCRA=0.98, P-score=0.98), followed by cigarette use (SUCRA=0.76, P-score=0.76), and former cigarette use (SUCRRA=0.50, P-score=0.49).

### Stroke

Four studies^26,30,32,33^ were included for pooled adjusted ORs (Supplementary file Figure 2). Cigarette and dual use were significantly associated with stroke, when compared to never use with pooled AORs (95% CI) of 1.84 (1.29–2.63) and 2.11 (1.41–3.15), respectively. However, the odds of stroke were not significantly higher for cigarette and dual users than those of e-cigarette users (Table 2). In addition, the odds of stroke in e-cigarette users did not differ significantly compared to non-users, with an AOR (95% CI) of 1.44 (0.92–2.24). According to SUCRA and P-score, dual use ranked highest for stroke risk (SUCRA=0.92 and P-score=0.93), followed by cigarette use (SUCRA=0.77 and P-score=0.76), e-cigarette use (SUCRA=0.45 and P-score=0.45), and former cigarette smoking (SUCRA=0.28 and P-score=0.29).

### Inconsistency, transitivity, and publication bias

Study characteristics across all those included were comparable (Table 1), suggesting the transitivity assumption was unlikely to be violated. Global *τ*^2^ and I^2^ were 0.037 and 94.6% for composite CVD, 0.052 and 45.6% for MI, and 0.089 and 64.6% for stroke, respectively. These values indicate high heterogeneity for the composite CVD outcome and moderate heterogeneity for MI and stroke outcomes, corresponding with Q statistics (P-value) of 260.45 (<0.001), 5.52 (0.138), and 22.57 (0.004), respectively. No evidence of inconsistency was identified by the design-by-treatment interaction model for adjusted effect pooling of the composite CVD, MI, and stroke outcomes with P-values of 0.575, 0.138, and 0.286, respectively. In addition, node-splitting using a back-calculation method did not indicate local inconsistency for almost all the comparisons (Supplementary file Table 8). No asymmetrical pattern was observed in the comparison-adjusted funnel plots for all outcomes, suggesting no publication bias (Supplementary file Figures 3–5). The Egger’s test P-values were 0.512, 0.123 and 0.876 for the composite CVD, MI, and stroke outcomes, respectively.

### Confidence in network meta-analysis

The confidence of evidence synthesis from the NMA was rated as low to very low for most comparisons across all outcomes (Supplementary file Table 9). The primary reason for downgrading was within-study bias.

## DISCUSSION

This SR and NMA evaluated associations between e-cigarette use and the risk of composite CVD events, MI, and stroke. Findings from the NMA suggested that e-cigarette use was significantly associated with an increased risk of composite CVD outcomes but was not associated with risk of MI and stroke, when compared to non-users. Consistent with the established evidence, our study confirmed significant increased risk of composite CVD, MI, and stroke in cigarette users. Additionally, dual use of e-cigarettes and cigarettes was also associated with significantly increased risk of composite CVD, MI, and stroke, when compared to both e-cigarette use and non-users. With respect to former users, only previous use of cigarette, and not e-cigarette, was significantly associated with risk of MI and composite CVD events.

The detrimental effect of e-cigarettes on CVD outcomes may be as a consequence of several mechanisms^34^. E-cigarettes contain substances such as nicotine, glycerol, propylene glycol, flavorings, and metal particles, which can activate macrophages and cause endothelial dysfunction, leading to inflammation and oxidative stress. Nicotine also disrupts lipid metabolism, raising low-density lipoprotein (LDL) and triglyceride levels while lowering high-density lipoprotein (HDL). Additionally, e-cigarette users have also had reported increased platelet activation, potentially promoting atherosclerosis^34^. One recent meta-analysis^35^ suggested that e-cigarettes might impair endothelial function measured by flow-mediated vasodilation, although their findings were inconclusive, given a lack of statistical significance.

Our results support these hypotheses through the significant associations identified between e-cigarette use and composite CVD events. However, findings from a recent SR using pairwise meta-analysis^36^ presented conflicting results, showing no significant association between exclusive e-cigarette use and CVD risk compared to never users. In contrast, the effect of dual use of cigarettes and e-cigarettes was consistent with our findings, indicating a significant association with increased risk of CVD outcomes. However, this pairwise meta-analysis was subject to several limitations, particularly given the use of the same data across different outcomes in the same pooled analysis. For example, effect sizes for coronary heart disease, MI, and stroke from the same study (e.g. Falk et al.^30^) were all combined in a single analysis. Additionally, some data overlapped across included studies. In contrast, our NMA separately pooled effect sizes for each reported outcome, avoiding such overlap. In addition, the sample size for pooling in this pairwise meta-analysis was smaller than in our NMA, i.e. 5 (after survey database duplication removal) versus 6 studies.

In contrast to the composite CVD outcomes, our NMA did not find a significant association between e-cigarette use and the odds of MI or stroke. These findings notably contradict previous pairwise meta-analyses, which reported a significant association between e-cigarette use and MI^13,14^ as well as stroke^16^. This may be due to sample size, since our study did observe a trend toward an increased risk of stroke from e-cigarette use, consistent with the direction of previously reported findings for stroke^16^. The discrepancies between our results and previous studies may also stem from differences in study design. Previous analyses combined exclusive e-cigarette use with dual use of cigarettes and e-cigarettes, while our study analyzed these groups separately. Additionally, previous studies also included overlapping data in their analyses, whereas our study made efforts to avoid such overlap when pooling data for each outcome.

Since the U.S. Food and Drug Administration (FDA) has authorized e-cigarettes for market entry to promote smoking cessation, harm perceptions of e-cigarettes have changed over time. Recent surveys^37,38^ demonstrated an increase in the proportion of adults, both smokers and non-smokers, perceiving that e-cigarettes were equally or more harmful than cigarettes. This phenomenon was possibly explained by an emergence of the e-cigarette or vaping use-associated lung injury in combination with an increased trend of youth vaping^38^. Our findings suggest that e-cigarettes still increase CVD risk (compared to no smoking) but do so less than cigarettes. Hence, the risks and benefits of e-cigarettes require accurate estimation and communication. Even though e-cigarettes can be used to promote cigarette cessation, they can also encourage the intention to smoke cigarettes^39^. Given the potential risks associated with e-cigarettes, policy should discourage new e-cigarette use in adolescents and adults that are never smokers. Fortunately, FDA-authorized e-cigarettes for market entry did not encourage e-cigarette use in these populations^40^. Nevertheless, product appeal (i.e. package and flavoring appealing to younger people) should be closely monitored^40^. In addition, several interventions, tailored to needs and user profiles, are required to promote e-cigarette cessation^41^.

This NMA investigated associations across various tobacco product exposures. The analysis utilized data from six distinct databases, and efforts were made to minimize overlap between study data to avoid including the same individuals more than once when pooling results. However, since most of the data originated from U.S. surveys, there remains a possibility that some individuals participated in more than one survey. Additionally, data from other regions, particularly Asian countries, are needed to better generalize the findings regarding the effects of e-cigarette use on the risk of CVD. Moreover, the number of included studies was relatively low which could be a reason for some non-significant associations.

### Limitations

There were some other limitations that should be acknowledged. First, most of the included studies relied on participants’ self-reported information regarding tobacco exposure and clinical outcomes that might be subject to misclassification and inaccurate reporting of both exposure and outcomes. These issues might introduce bias to the study findings. Second, the covariates considered in the multivariate analysis varied across individual studies, meaning that the adjusted OR may differ between studies. Moreover, this study is not an individual patient meta-analysis, where effect pooling can be performed using a statistical model that incorporates a uniform set of covariates. Third, this study did not assess the effects of different types of tobacco products due to the limited number of included studies and the lack of data for direct comparisons. Lastly, because the data were primarily derived from cross-sectional studies, the analysis can only identify associations and cannot infer the causal relationships between e-cigarettes and CVD. In addition, it is also possible that patients switched from cigarettes to e-cigarette use following a CVD diagnosis.

## CONCLUSIONS

Our analysis found that current use of e-cigarettes and cigarettes was significantly associated with a greater risk of composite CVD outcomes. However, only cigarette use and dual use were significantly associated with MI and stroke when both outcomes were evaluated independently. Additionally, only former use of cigarettes, but not e-cigarettes, was associated with an increased risk of composite CVD events. However, given that most studies were limited by their cross-sectional design, the temporality of a causal relationship between e-cigarette use and CVD risk cannot be established. Therefore, evidence from prospective cohort studies is required to confirm these findings.

## Supplementary Material



## Data Availability

The data supporting this research can be found in the Supplementary file.
