# Supplementary material for: Electronic cigarettes and cardiovascular diseases: An updated systematic review and network meta-analysis
Source: Tob Induc Dis. 2025 Sep 5;23:10.18332/tid/208065. doi: 10.18332/tid/208065 (PMC12412302; doi:10.18332/tid/208065)
Supplement: Supplementary file 1 [file TID-23-124-s1.pdf]

Supplementary Material 1.

Section and Topic	Item #	Checklist item	Location where item is reported
**TITLE**			
Title	1	Identify the report as a systematic review.	Title page
**ABSTRACT**			
Abstract	2	See the PRISMA 2020 for Abstracts checklist.	P4
**INTRODUCTION**			
Rationale	3	Describe the rationale for the review in the context of existing knowledge.	P6
Objectives	4	Provide an explicit statement of the objective(s) or question(s) the review addresses.	P7
**METHODS**			
Eligibility criteria	5	Specify the inclusion and exclusion criteria for the review and how studies were grouped for the syntheses.	P8
Information sources	6	Specify all databases, registers, websites, organisations, reference lists and other sources searched or consulted to identify studies. Specify the date when each source was last searched or consulted.	P7
Search strategy	7	Present the full search strategies for all databases, registers and websites, including any filters and limits used.	Appendix
Selection process	8	Specify the methods used to decide whether a study met the inclusion criteria of the review, including how many reviewers screened each record and each report retrieved, whether they worked independently, and if applicable, details of automation tools used in the process.	P7-8
Data collection process	9	Specify the methods used to collect data from reports, including how many reviewers collected data from each report, whether they worked independently, any processes for obtaining or confirming data from study investigators, and if applicable, details of automation tools used in the process.	P9
Data items	10a	List and define all outcomes for which data were sought. Specify whether all results that were compatible with each outcome domain in each study were sought (e.g. for all measures, time points, analyses), and if not, the methods used to decide which results to collect.	P8-9
	10b	List and define all other variables for which data were sought (e.g. participant and intervention characteristics, funding sources). Describe any assumptions made about any missing or unclear information.	P9
Study risk of bias assessment	11	Specify the methods used to assess risk of bias in the included studies, including details of the tool(s) used, how many reviewers assessed each study and whether they worked independently, and if applicable, details of automation tools used in the process.	P9-10
Effect measures	12	Specify for each outcome the effect measure(s) (e.g. risk ratio, mean difference) used in the synthesis or presentation of results.	P10
Synthesis methods	13a	Describe the processes used to decide which studies were eligible for each synthesis (e.g. tabulating the study intervention characteristics and comparing against the planned groups for each synthesis (item #5)).	P10
	13b	Describe any methods required to prepare the data for presentation or synthesis, such as handling of missing summary statistics, or data conversions.	P10

Section and Topic	Item #	Checklist item	Location where item is reported
	13c	Describe any methods used to tabulate or visually display results of individual studies and syntheses.	P10
	13d	Describe any methods used to synthesize results and provide a rationale for the choice(s). If meta-analysis was performed, describe the model(s), method(s) to identify the presence and extent of statistical heterogeneity, and software package(s) used.	P10
	13e	Describe any methods used to explore possible causes of heterogeneity among study results (e.g. subgroup analysis, meta-regression).	P10
	13f	Describe any sensitivity analyses conducted to assess robustness of the synthesized results.	N/A
Reporting bias assessment	14	Describe any methods used to assess risk of bias due to missing results in a synthesis (arising from reporting biases).	P10
Certainty assessment	15	Describe any methods used to assess certainty (or confidence) in the body of evidence for an outcome.	P10
**RESULTS**			
Study selection	16a	Describe the results of the search and selection process, from the number of records identified in the search to the number of studies included in the review, ideally using a flow diagram.	P11<br>Figure 1
	16b	Cite studies that might appear to meet the inclusion criteria, but which were excluded, and explain why they were excluded.	Appendix
Study characteristics	17	Cite each included study and present its characteristics.	P11-12<br>Table 1
Risk of bias in studies	18	Present assessments of risk of bias for each included study.	P12<br>Appendix
Results of individual studies	19	For all outcomes, present, for each study: (a) summary statistics for each group (where appropriate) and (b) an effect estimate and its precision (e.g. confidence/credible interval), ideally using structured tables or plots.	Supplement
Results of syntheses	20a	For each synthesis, briefly summarise the characteristics and risk of bias among contributing studies.	Appendix
	20b	Present results of all statistical syntheses conducted. If meta-analysis was done, present for each the summary estimate and its precision (e.g. confidence/credible interval) and measures of statistical heterogeneity. If comparing groups, describe the direction of the effect.	P12-14<br>Table 2
	20c	Present results of all investigations of possible causes of heterogeneity among study results.	P14-15
	20d	Present results of all sensitivity analyses conducted to assess the robustness of the synthesized results.	N/A
Reporting biases	21	Present assessments of risk of bias due to missing results (arising from reporting biases) for each synthesis assessed.	P15<br>Appendix
Certainty of evidence	22	Present assessments of certainty (or confidence) in the body of evidence for each outcome assessed.	P15<br>Appendix
**DISCUSSION**			
Discussion	23a	Provide a general interpretation of the results in the context of other evidence.	P15-17
	23b	Discuss any limitations of the evidence included in the review.	P18
	23c	Discuss any limitations of the review processes used.	P18
	23d	Discuss implications of the results for practice, policy, and future research.	P19

Section and Topic	Item #	Checklist item	Location where item is reported
**OTHER INFORMATION**			
Registration and protocol	24a	Provide registration information for the review, including register name and registration number, or state that the review was not registered.	P3
	24b	Indicate where the review protocol can be accessed, or state that a protocol was not prepared.	P3
	24c	Describe and explain any amendments to information provided at registration or in the protocol.	N/A
Support	25	Describe sources of financial or non-financial support for the review, and the role of the funders or sponsors in the review.	P3
Competing interests	26	Declare any competing interests of review authors.	P3
Availability of data, code and other materials	27	Report which of the following are publicly available and where they can be found: template data collection forms; data extracted from included studies; data used for all analyses; analytic code; any other materials used in the review.	P3<br>Supplement

Section and Topic	Item #	Checklist item	Reported (Yes/No)
**TITLE**			
Title	1	Identify the report as a systematic review.	Yes
**BACKGROUND**			
Objectives	2	Provide an explicit statement of the main objective(s) or question(s) the review addresses.	Yes
**METHODS**			
Eligibility criteria	3	Specify the inclusion and exclusion criteria for the review.	Yes
Information sources	4	Specify the information sources (e.g. databases, registers) used to identify studies and the date when each was last searched.	Yes
Risk of bias	5	Specify the methods used to assess risk of bias in the included studies.	Yes
Synthesis of results	6	Specify the methods used to present and synthesise results.	Yes
**RESULTS**			
Included studies	7	Give the total number of included studies and participants and summarise relevant characteristics of studies.	Yes
Synthesis of results	8	Present results for main outcomes, preferably indicating the number of included studies and participants for each. If meta-analysis was done, report the summary estimate and confidence/credible interval. If comparing groups, indicate the direction of the effect (i.e. which group is favoured).	Yes

Section and Topic	Item #	Checklist item	Reported (Yes/No)
**DISCUSSION**			
Limitations of evidence	9	Provide a brief summary of the limitations of the evidence included in the review (e.g. study risk of bias, inconsistency and imprecision).	Yes
Interpretation	10	Provide a general interpretation of the results and important implications.	Yes
**OTHER**			
Funding	11	Specify the primary source of funding for the review.	Yes
Registration	12	Provide the register name and registration number.	Yes

From: Page MJ, McKenzie JE, Bossuyt PM, Boutron I, Hoffmann TC, Mulrow CD, et al. The PRISMA 2020 statement: an updated guideline for reporting systematic reviews. BMJ 2021;372:n71. doi: 10.1136/bmj.n71. This work is licensed under CC BY 4.0. To view a copy of this license, visit https://creativecommons.org/licenses/by/4.0/

Supplementary Table 1. Search terms and strategy for the primary search

Database	Search terms	Number of identified records
Medline	((E-cigarette) OR ("Electronic cigarette") OR (E-cigarettes) OR ("Electronic cigarettes") OR ("Electronic nicotine de*") OR ("Electronic nicotine delivery system") OR (Vape) OR ("Electronic inhalant device") OR ("Vaping"[Mesh]) OR ("Electronic Nicotine Delivery Systems"[Mesh]))	439
Scopus	((TITLE-ABS-KEY (e-cigarette)) OR (TITLE-ABS-KEY ("electronic cigarette")) OR (TITLE-ABS-KEY ("electronic nicotine de*")) OR (TITLE-ABS-KEY ("electronic nicotine delivery system")) OR (TITLE-ABS-KEY (vape)) OR (TITLE-ABS-KEY ("electronic inhalant device")))	547

Supplementary Table 2. Search terms and strategies for the secondary search

Database	Search terms	Number of identified records
Medline	((((((((((E-cigarette) OR ("Electronic cigarette")) OR (E-cigarettes)) OR ("Electronic cigarettes")) OR ("Electronic nicotine de*")) OR ("Electronic nicotine delivery system")) OR (Vape)) OR ("Electronic inhalant device")) OR ("Vaping"[Mesh])) OR ("Electronic Nicotine Delivery Systems"[Mesh]))) AND ((((((((cardiovascular) OR (coronary)) OR (cerebrovascular)) OR (cerebral)) OR (carotid)) AND ((((disease) OR (syndrome)) OR (accident)) OR (stenosis))) OR (((("myocardial infarction") OR (angina)) OR (stroke)) OR ("transient ischemic attack"))) OR ((((((coronary stenosis[MeSH Terms]) OR (coronary disease[MeSH Terms])) OR (coronary artery disease[MeSH Terms])) OR (myocardial infarction[MeSH Terms])) OR (acute coronary syndrome[MeSH Terms])) OR (((cerebrovascular disorder[MeSH Terms]) OR (stroke[MeSH Terms])) OR (Ischemic Attack, Transient[MeSH Terms]))))	204
Scopus	((TITLE-ABS-KEY (e-cigarette)) OR (TITLE-ABS-KEY ("electronic cigarette")) OR (TITLE-ABS-KEY ("electronic nicotine de*")) OR (TITLE-ABS-KEY ("electronic nicotine delivery system")) OR (TITLE-ABS-KEY (vape)) OR (TITLE-ABS-KEY ("electronic inhalant device"))) AND (((TITLE-ABS-KEY (cardiovascular OR coronary OR cerebrovascular OR cerebral OR carotid)) AND (TITLE-ABS-KEY (disease OR syndrome OR accident OR stenosis))) OR ((TITLE-ABS-KEY ("myocardial infarction")) OR (TITLE-ABS-KEY (angina)) OR (TITLE-ABS-KEY (stroke)) OR (TITLE-ABS-KEY ("transient ischemic attack")))) AND PUBYEAR > 2019 AND PUBYEAR < 2025 AND (LIMIT-TO (DOCTYPE, "ar") OR LIMIT-TO (DOCTYPE, "cp"))	503

Supplementary Table 3. Exposure definition for the included studies

Author, Year	Exposure	Definition
Wang 2018	E-cigarette use	"Yes" to the question "Do you now use e-cigarettes?"
	Cigarette use	"Yes" to the question "Do you smoke now?"
	Dual use	"Yes" to both questions
	Never use	"No" to both questions
Farsalinos 2019	E-cigarette use	"Yes" to the question "Have you ever use an e-cigarette even one time?" and "Yes" to the question "Do you now use e-cigarettes?"
	Former E-cigarette use	"Yes" to the question "Have you ever use an e-cigarette even one time?" and "No" to the question "Do you now use e-cigarettes?"
	Never use	"No" to the question "Have you ever use an e-cigarette even one time?"
Osei 2019	E-cigarette use	"Yes" to the question "Have you ever used an e-cigarette or other electronic vaping product, even just one time, in your entire life?" and "Yes" to the question "Do you now use e-cigarettes or other electronic vaping products?" and "No" to the question "Have you smoked at least 100 cigarettes in your entire life?"
	Never use	"No" to the question "Have you ever used an e-cigarette or other electronic vaping product, even just one time, in your entire life?" and "No" to the question "Have you smoked at least 100 cigarettes in your entire life?"
Parekh 2020	E-cigarette use	"Yes" to the question "Have you ever used an e-cigarette or other electronic vaping product, even just one time, in your entire life?" and "Yes" to the question "Do you now use e-cigarettes or other electronic vaping products?"
	Cigarette use	"Yes" to the question "Have you smoked at least 100 cigarettes in your entire life?" and "Yes" to the question "Do you now smoke?"
	Dual use	Meet both criteria for E-cigarette and cigarette use
	Never use	"No" to the question "Have you ever used an e-cigarette or other electronic vaping product, even just one time, in your entire life?" and "No" to the question "Have you smoked at least 100 cigarettes in your entire life?"
Choi 2021	E-cigarette use	Did not provide key questions for categorization.
	Cigarette use	
	Dual use	
	Former cigarette use	
	Never use	
Critcher 2021	E-cigarette use	"Yes" to the question "Have you ever use an e-cigarette even one time?" and "Yes" to the question "Do you now use e-cigarettes?"
	Former E-cigarette use	"Yes" to the question "Have you ever use an e-cigarette even one time?" and "No" to the question "Do you now use e-cigarettes?"
	Never use	"No" to the question "Have you ever use an e-cigarette even one time?"
Berlowitz 2022	E-cigarette use	"Yes" if currently use e-cigarettes
	Cigarette use	"Yes" if > 100 cigarettes smoking in their entire life and currently smoking

Author, Year	Exposure	Definition
	Dual use	“Yes” to both conditions
	Never use	“No” to both conditions
Falk 2022	E-cigarette use	“Yes” to the question “Have you ever use an e-cigarette even one time?” and “Yes” to the question “Do you now use e-cigarettes?”
	Cigarette use	“Yes” to the question “Have you smoke at least 100 cigarettes in your entire life?” and “Yes” to the question “Do you now smoke?”
	Dual use	Meet both criteria for E-cigarette and cigarette use
	Former cigarette use	“Yes” to the question “Have you smoke at least 100 cigarettes in your entire life?” and “No” to the question “Do you now smoke?”
	Never use	“No” to the question “Have you ever use an e-cigarette even one time?” and “No” to the question “Have you smoke at least 100 cigarettes in your entire life?”
Liu 2022	E-cigarette use	“Yes” to the question “Have you ever used an e-cigarette or other electronic vaping product, even just one time, in your entire life?” and “Yes” to the question “Do you now use e-cigarettes or other electronic vaping products?”
	Cigarette use	“Yes” to the question “Have you smoked at least 100 cigarettes in your entire life?” and “Yes” to the question “Do you now smoke?”
	Dual use	Meet both criteria for E-cigarette and cigarette use
	Former E-cigarette use	“Yes” to the question “Have you ever used an e-cigarette or other electronic vaping product, even just one time, in your entire life?” and “No” to the question “Do you now use e-cigarettes or other electronic vaping products?”
	Former cigarette use	“Yes” to the question “Have you smoked at least 100 cigarettes in your entire life?” and “No” to the question “Do you now smoke?”
	Former dual use	Meet both criteria for former E-cigarette and cigarette use
	Never use	“No” to the question “Have you ever used an e-cigarette or other electronic vaping product, even just one time, in your entire life?” and “No” to the question “Have you smoked at least 100 cigarettes in your entire life?”
Patel 2022	E-cigarette use	“Yes” to the question “Ever used an e-cigarette?” and “During the past 30 days?”
	Cigarette use	“Yes” to the question “Smoked at least 100 cigarettes in life?”
	Dual use	Meet both conditions
Hirschtick 2023	E-cigarette use	“Yes” if currently use e-cigarettes
	Cigarette use	“Yes” if > 100 cigarettes smoking in their entire life and currently smoking
	Dual use	“Yes” to both conditions
	Never use	“No” to both conditions

E-cigarette, electronic cigarette

Supplementary Table 4. Definition of outcome of the included studies

Author, Year	Outcome	Definition
Wang 2018	MI	Participant report of heart attack told by health professional
	Stroke	Participant report of stroke told by health professional
	Composite CVD	Participant report of coronary artery disease or stroke told by health professional
Farsalinos 2019	Composite CVD	Participant report of coronary heart disease told by health professional
Osei 2019	Composite CVD	Participant report of coronary heart disease, MI, or stroke told by health professional
Parekh 2020	Stroke	Participant report of stroke told by health professional
Choi 2021	Composite CVD	ICD-10 (I20-I25 and I60-I69)
Critcher 2021	MI	Participant report of MI told by health professional
Berlowitz 2022	Composite CVD	Participant report of MI, bypass surgery, heart failure, other heart condition, or stroke told by health professional
Falk 2022	MI	Participant report of MI told by health professional
	Stroke	Participant report of stroke told by health professional
	Composite CVD	Participant report of coronary artery disease told by health professional
Liu 2022	Composite CVD	Participant report of coronary heart disease, heart attack, or stroke told by health professional
Patel 2022	Stroke	Participant report of stroke told by health professional
	Composite CVD	Participant report of coronary heart disease told by health professional
Hirschtick 2023	MI	Participant report of heart attack or needed bypass surgery told by health professional
	Stroke	Participant report of stroke told by health professional

CVD, cardiovascular disease; ICD-10, international classification of diseases- 10th revision; MI, myocardial infarction

Supplementary Table 5. Covariates that were considered in the multivariate models used for estimating the adjusted odds ratio of exposure

Author Year	Adjusting covariate
Osei 2019	age, sex, race, education, income, physical activity, BMI, alcohol
Parekh 2020	age, sex, race, education, income, marital status, insurance, physical activity, alcohol, DM, HT, cholesterol
Choi 2021	age, income, employment, area of residence, alcohol, physical activity, BMI, SBP, FSG, drug abuse, Charlson comorbidity index
Berlowitz 2022	age, sex, race, education, BMI, Marijuana, HT, DM, cholesterol, family history of CAD
Falk 2022	age, sex, BMI
Liu 2022	age, sex, race, education, physical activity, chewing tobacco, BMI, DM, COPD, depression
Patel 2023	age, sex, BMI, income, HT, DM, cholesterol, depression, cancer, substance abuse, aspirin use
Hirschtick 2023	age, sex, race, education, HT, DM, family history of premature MI

BMI, body mass index; CAD, coronary artery disease; COPD, chronic obstructive pulmonary disease; DM, diabetes mellitus; FSG, fasting serum glucose; HT, hypertension; MI, myocardial infarction; SBP, systolic blood pressure

Supplementary Table 6. Risk of bias assessment of cross-sectional studies

	Wang 2018	Farsalinos 2019	Osei 2019	Parekh 2020	Choi 2021	Critcher 2021	Falk 2022	Liu 2022	Patel 2022
Were the criteria for inclusion in the sample clearly defined?	Y	Y	Y	Y	Y	Y	Y	Y	Y
Were the study subjects and the setting described in detail?	N	Y	Y	Y	Y	Y	Y	Y	Y
Was the exposure measured in a valid and reliable way?	Y	Y	Y	Y	Y	Y	Y	Y	Y
Were objective, standard criteria used for measurement of the condition?	Y	Y	Y	Y	Y	Y	Y	Y	Y
Were confounding factors identified?	Y	Y	Y	Y	Y	Y	Y	Y	Y
Were strategies to deal with confounding factors stated?	Y	Y	Y	Y	Y	Y	Y	Y	Y
Were the outcomes measured in a valid and reliable way?	N	N	N	N	Y	N	N	N	N
Was appropriate statistical analysis used?	Y	Y	Y	Y	Y	Y	Y	Y	Y

Supplementary Table 7. Risk of bias assessment of included cohort studies

Studies	Selection				Comparability	Outcome			Overall quality
	Representativeness	Selection of the non exposed cohort	Ascertainment of exposure	Outcome of interest not present at start of study		Outcome assessment	Follow-up long enough	Adequacy of follow up	
**Berlowitz 2022**	A (*)	A (*)	C	B	**	C	B	D	Poor
**Hirschtick 2023**	A (*)	A (*)	C	B	**	C	B	D	Poor

Thresholds for converting the Newcastle-Ottawa scales to AHRQ standards (good, fair, and poor):
Good quality: 3 or 4 stars in selection domain AND 1 or 2 stars in comparability domain AND 2 or 3 stars in outcome/exposure domain
Fair quality: 2 stars in selection domain AND 1 or 2 stars in comparability domain AND 2 or 3 stars in outcome/exposure domain
Poor quality: 0 or 1 star in selection domain OR 0 stars in comparability domain OR 0 or 1 stars in outcome/exposure domain

Supplementary Table 8. Local consistency network checkusing a node-splitting approach

Comparison	P-value		
	Composite CVD	MI	Stroke
Dual:Cigarette	0.981	0.240	0.586
Dual:E-cigarette	0.831	0.314	0.726
Dual:Former Dual	0.588	-	-
Dual:Former Cigarette	0.324	0.334	0.581
Dual:Former E-cigarette	0.588	-	-
Dual:Never Use	0.274	0.442	0.403
Cigarette:E-cigarette	0.969	0.442	0.598
Cigarette:Former Dual	0.246	-	-
Cigarette:Former Cigarette	0.374	0.508	0.543
Cigarette:Former E-cigarette	0.246	-	-
Cigarette:Never Use	0.154	0.314	0.317
E-cigarette:Former Dual	0.922	-	-
E-cigarette:Former Cigarette	0.401	0.749	0.253
E-cigarette:Former E-cigarette	0.922	-	-
E-cigarette:Never Use	0.014	0.240	0.031
Former Dual:Former Cigarette	0.560	-	-
Former Dual:Former E-cigarette	-	-	-
Former Dual:Never Use	0.839	-	-
Former Cigarette:Former E-cigarette	0.560	-	-
Former Cigarette:Never Use	0.392	0.200	0.7510
Former E-cigarette:Never Use	0.839	-	-

CVD, cardiovascular disease; MI, myocardial infarction

Supplementary Table 9. Network meta-analysis confidence rating check

Composite cardiovascular outcomes									
Comparison	***Number of studies***	***Within-study bias***	***Reporting bias***	***Indirectness***	***Imprecision***	***Heterogeneity***	***Incoherence***	***Confidence rating***	***Reason(s) for downgrading***
Never Use: Former E-cigarette	1	Major concerns	Low risk	No concerns	Some concerns	No concerns	No concerns	Very low	Within-study bias, Imprecision
Never Use: Former Cigarette	3	Major concerns	Low risk	No concerns	No concerns	No concerns	No concerns	Low	Within-study bias
Never Use: Former Dual	1	Major concerns	Low risk	No concerns	No concerns	No concerns	No concerns	Low	Within-study bias
Never Use: E-cigarette	5	Major concerns	Low risk	No concerns	No concerns	No concerns	Some concerns	Very low	Within-study bias, Incoherence
Never Use: Cigarette	4	Major concerns	Low risk	No concerns	No concerns	No concerns	No concerns	Low	Within-study bias
Never Use: Dual	4	Major concerns	Low risk	No concerns	No concerns	No concerns	No concerns	Low	Within-study bias
Former E-cigarette: Former Cigarette	1	Major concerns	Low risk	No concerns	No concerns	No concerns	No concerns	Low	Within-study bias
Former E-cigarette: Former Dual	1	Major concerns	Low risk	No concerns	No concerns	No concerns	No concerns	Low	Within-study bias
Former E-cigarette: E-cigarette	1	Major concerns	Low risk	No concerns	No concerns	No concerns	No concerns	Low	Within-study bias
Former E-cigarette: Cigarette	1	Major concerns	Low risk	No concerns	No concerns	No concerns	No concerns	Low	Within-study bias
Former E-cigarette: Dual	1	Major concerns	Low risk	No concerns	No concerns	No concerns	No concerns	Low	Within-study bias
Former Cig: Former Dual	1	Major concerns	Low risk	No concerns	No concerns	Some concerns	No concerns	Very low	Within-study bias, Heterogeneity
Former Cigarette: E-cigarette	3	Major concerns	Low risk	No concerns	No concerns	No concerns	Major concerns	Very low	Within-study bias, Incoherence
Former Cigarette: Cigarette	3	Major concerns	Low risk	No concerns	No concerns	No concerns	No concerns	Low	Within-study bias
Former Cigarette: Dual	3	No concerns	Low risk	No concerns	No concerns	No concerns	No concerns	High	–
Former Dual: E-cigarette	1	Major concerns	Low risk	No concerns	No concerns	No concerns	No concerns	Low	Within-study bias
Former Dual: Cigarette	1	Major concerns	Low risk	No concerns	No concerns	Some concerns	No concerns	Very low	Within-study bias, Heterogeneity

Former Dual: Dual	1	Major concerns	Low risk	No concerns	No concerns	Some concerns	No concerns	Very low	Within-study bias, Heterogeneity
E-cigarette: Cigarette	5	Major concerns	Low risk	No concerns	No concerns	No concerns	Some concerns	Very low	Within-study bias, Incoherence
E-cigarette: Dual	5	Major concerns	Low risk	No concerns	No concerns	No concerns	No concerns	Low	Within-study bias
Cigarette: Dual	5	Major concerns	Low risk	No concerns	No concerns	No concerns	No concerns	Low	Within-study bias
**Myocardial infarction**									
Comparison	***Number of studies***	***Within-study bias***	***Reporting bias***	***Indirectness***	***Imprecision***	***Heterogeneity***	***Incoherence***	***Confidence rating***	***Reason(s) for downgrading***
Never Use: Former Cigarette	1	Major concerns	Low risk	No concerns	No concerns	No concerns	No concerns	Low	Within-study bias
Never Use: E-cigarette	2	Major concerns	Low risk	No concerns	Major concerns	No concerns	No concerns	Very low	Within-study bias, Imprecision
Never Use: Cigarette	2	Major concerns	Low risk	No concerns	No concerns	No concerns	No concerns	Low	Within-study bias
Never Use: Dual	2	Major concerns	Low risk	No concerns	No concerns	No concerns	No concerns	Low	Within-study bias
Former Cigarette: E-cigarette	1	Major concerns	Low risk	No concerns	No concerns	No concerns	No concerns	Low	Within-study bias
Former Cigarette: Cigarette	1	Major concerns	Low risk	No concerns	No concerns	No concerns	No concerns	Low	Within-study bias
Former Cigarette: Dual	1	Major concerns	Low risk	No concerns	No concerns	No concerns	No concerns	Low	Within-study bias
E-cigarette: Cigarette	2	Major concerns	Low risk	No concerns	No concerns	No concerns	No concerns	Low	Within-study bias
E-cigarette: Dual	2	Major concerns	Low risk	No concerns	No concerns	No concerns	No concerns	Low	Within-study bias
Cigarette: Dual	2	Major concerns	Low risk	No concerns	No concerns	No concerns	No concerns	Low	Within-study bias
**Stroke**									
Comparison	***Number of studies***	***Within-study bias***	***Reporting bias***	***Indirectness***	***Imprecision***	***Heterogeneity***	***Incoherence***	***Confidence rating***	***Reason(s) for downgrading***
Never Use: Former Cigarette	1	Major concerns	Low risk	No concerns	No concerns	No concerns	No concerns	Low	Within-study bias
Never Use: E-cigarette	3	Major concerns	Low risk	No concerns	No concerns	No concerns	Major concerns	Very low	Within-study bias, Incoherence
Never Use: Cigarette	3	Major concerns	Low risk	No concerns	No concerns	No concerns	No concerns	Low	Within-study bias

Never Use: Dual	3	Major concerns	Low risk	No concerns	No concerns	No concerns	No concerns	Low	Within-study bias
Former Cigarette: E-cigarette	1	Major concerns	Low risk	No concerns	No concerns	No concerns	Major concerns	Very low	Within-study bias, Incoherence
Former Cigarette: Cigarette	1	Major concerns	Low risk	No concerns	No concerns	No concerns	No concerns	Low	Within-study bias
Former Cigarette: Dual	1	Major concerns	Low risk	No concerns	No concerns	No concerns	No concerns	Low	Within-study bias
E-cigarette: Cigarette	4	Major concerns	Low risk	No concerns	No concerns	No concerns	No concerns	Low	Within-study bias
E-cigarette: Dual	4	Major concerns	Low risk	No concerns	No concerns	No concerns	No concerns	Low	Within-study bias
Cigarette: Dual	4	Major concerns	Low risk	No concerns	No concerns	No concerns	No concerns	Low	Within-study bias

Supplementary Table 10.

CVD

ID	Author	Year	_design	expcode2	expcode1	lnOR	SE	_contrast
4	Osei1	2019	0 4	0	4	0.0392	0.2562	4 - 0
8	Choi7	2021	0 2 4 5 6	0	2	0.1363	0.0214	2 - 0
8	Choi7	2021	0 2 4 5 6	0	4	0.3378	0.1403	4 - 0
8	Choi7	2021	0 2 4 5 6	0	5	0.6005	0.0138	5 - 0
8	Choi7	2021	0 2 4 5 6	0	6	0.4132	0.0309	6 - 0
8	Choi7	2021	0 2 4 5 6	2	4	0.2015	0.1405	4 - 2
8	Choi7	2021	0 2 4 5 6	2	5	0.4642	0.0163	5 - 2
8	Choi7	2021	0 2 4 5 6	2	6	0.2769	0.0321	6 - 2
8	Choi7	2021	0 2 4 5 6	4	5	0.2627	0.1396	5 - 4
8	Choi7	2021	0 2 4 5 6	4	6	0.0754	0.1423	6 - 4
8	Choi7	2021	0 2 4 5 6	5	6	-0.1873	0.0277	6 - 5
10	Berlowitz4	2022	0 4 5 6	0	4	0.0000	0.1993	4 - 0
10	Berlowitz4	2022	0 4 5 6	0	5	0.4525	0.0881	5 - 0
10	Berlowitz4	2022	0 4 5 6	0	6	0.4595	0.1330	6 - 0
10	Berlowitz4	2022	0 4 5 6	4	5	0.4525	0.2179	5 - 4
10	Berlowitz4	2022	0 4 5 6	4	6	0.4595	0.2396	6 - 4
10	Berlowitz4	2022	0 4 5 6	5	6	0.0070	0.1596	6 - 5
12	Falk10	2022	0 2 4 5 6	0	2	0.4259	0.0449	2 - 0
12	Falk10	2022	0 2 4 5 6	0	4	-0.1555	0.2553	4 - 0
12	Falk10	2022	0 2 4 5 6	0	5	0.6217	0.0737	5 - 0
12	Falk10	2022	0 2 4 5 6	0	6	0.7934	0.0944	6 - 0
12	Falk10	2022	0 2 4 5 6	2	4	-0.5814	0.2592	4 - 2
12	Falk10	2022	0 2 4 5 6	2	5	0.1957	0.0864	5 - 2
12	Falk10	2022	0 2 4 5 6	2	6	0.3675	0.1046	6 - 2
12	Falk10	2022	0 2 4 5 6	4	5	0.7771	0.2658	5 - 4
12	Falk10	2022	0 2 4 5 6	4	6	0.9489	0.2722	6 - 4
12	Falk10	2022	0 2 4 5 6	5	6	0.1718	0.1198	6 - 5
13	Liu14	2022	0 1 2 3 4 5 6	0	1	-0.1416	0.1411	1 - 0

13	Liu14	2022	0 1 2 3 4 5 6	0	2	0.2912	0.0370	2 - 0
13	Liu14	2022	0 1 2 3 4 5 6	0	3	0.3591	0.0791	3 - 0
13	Liu14	2022	0 1 2 3 4 5 6	0	4	0.2231	0.2270	4 - 0
13	Liu14	2022	0 1 2 3 4 5 6	0	5	0.2979	0.0580	5 - 0
13	Liu14	2022	0 1 2 3 4 5 6	0	6	0.5811	0.1370	6 - 0
13	Liu14	2022	0 1 2 3 4 5 6	1	2	0.4327	0.1459	2 - 1
13	Liu14	2022	0 1 2 3 4 5 6	1	3	0.5006	0.1618	3 - 1
13	Liu14	2022	0 1 2 3 4 5 6	1	4	0.3647	0.2672	4 - 1
13	Liu14	2022	0 1 2 3 4 5 6	1	5	0.4394	0.1526	5 - 1
13	Liu14	2022	0 1 2 3 4 5 6	1	6	0.7227	0.1967	6 - 1
13	Liu14	2022	0 1 2 3 4 5 6	2	3	0.0679	0.0874	3 - 2
13	Liu14	2022	0 1 2 3 4 5 6	2	4	-0.0680	0.2300	4 - 2
13	Liu14	2022	0 1 2 3 4 5 6	2	5	0.0067	0.0688	5 - 2
13	Liu14	2022	0 1 2 3 4 5 6	2	6	0.2899	0.1419	6 - 2
13	Liu14	2022	0 1 2 3 4 5 6	3	4	-0.1359	0.2404	4 - 3
13	Liu14	2022	0 1 2 3 4 5 6	3	5	-0.0612	0.0981	5 - 3
13	Liu14	2022	0 1 2 3 4 5 6	3	6	0.2220	0.1582	6 - 3
13	Liu14	2022	0 1 2 3 4 5 6	4	5	0.0747	0.2343	5 - 4
13	Liu14	2022	0 1 2 3 4 5 6	4	6	0.3580	0.2651	6 - 4
13	Liu14	2022	0 1 2 3 4 5 6	5	6	0.2832	0.1488	6 - 5
15	Patel2	2022	4 5 6	4	5	-0.1398	0.0022	5 - 4
15	Patel2	2022	4 5 6	4	6	-0.0087	0.0031	6 - 4
15	Patel2	2022	4 5 6	5	6	0.1310	0.0022	6 - 5

Supplementary Table 11.

Treat Code

0	Never use
1	Former E-cig
2	Former Cig
3	Former Dual
4	E-cig
5	Cig
6	Dual

Supplementary Table 12.

MI

ID	Author	Year	_design	expose2	expose1	lnOR	SE	_contrast
12	Falk10	2022	0 2 4 5 6	Never Use	Former Cigarette	0.5608	0.0539	2 - 0
12	Falk10	2022	0 2 4 5 6	Never Use	E-cigarette	-0.0161	0.2925	4 - 0
12	Falk10	2022	0 2 4 5 6	Never Use	Cigarette	1.0424	0.0763	5 - 0
12	Falk10	2022	0 2 4 5 6	Never Use	Dual	1.3452	0.0878	6 - 0
12	Falk10	2022	0 2 4 5 6	Former Cigarette	E-cigarette	-0.5769	0.2975	4 - 2
12	Falk10	2022	0 2 4 5 6	Former Cigarette	Cigarette	0.4816	0.0935	5 - 2
12	Falk10	2022	0 2 4 5 6	Former Cigarette	Dual	0.7845	0.1030	6 - 2
12	Falk10	2022	0 2 4 5 6	E-cigarette	Cigarette	1.0585	0.3023	5 - 4
12	Falk10	2022	0 2 4 5 6	E-cigarette	Dual	1.3613	0.3054	6 - 4
12	Falk10	2022	0 2 4 5 6	Cigarette	Dual	0.3028	0.1164	6 - 5
17	Hirschtick6	2023	0 4 5 6	Never Use	E-cigarette	-0.4930	0.8221	4 - 0
17	Hirschtick6	2023	0 4 5 6	Never Use	Cigarette	0.6849	0.1792	5 - 0
17	Hirschtick6	2023	0 4 5 6	Never Use	Dual	0.6070	0.5354	6 - 0
17	Hirschtick6	2023	0 4 5 6	E-cigarette	Cigarette	1.1779	0.8414	5 - 4

17	Hirschtick6	2023	0 4 5 6	E-cigarette	Dual	1.1000	0.9810	6 - 4
17	Hirschtick6	2023	0 4 5 6	Cigarette	Dual	-0.0779	0.5646	6 - 5

Supplementary Table 13.

Stroke

ID	Author	Year	_design	expose2	expose1	lnOR	SE	_contrast
5	Parekh 4	2020	0 4 5 6	Never Use	E-cigarette	-0.3711	0.3647	4 - 0
5	Parekh 4	2020	0 4 5 6	Never Use	Cigarette	0.4637	0.1700	5 - 0
5	Parekh 4	2020	0 4 5 6	Never Use	Dual	1.0682	0.2999	6 - 0
5	Parekh 4	2020	0 4 5 6	E-cigarette	Cigarette	0.8348	0.4023	5 - 4
5	Parekh 4	2020	0 4 5 6	E-cigarette	Dual	1.4392	0.4722	6 - 4
5	Parekh 4	2020	0 4 5 6	Cigarette	Dual	0.6044	0.3448	6 - 5
12	Falk5	2022	0 2 4 5 6	Never Use	Former Cigarette	0.2523	0.0564	2 - 0
12	Falk5	2022	0 2 4 5 6	Never Use	E-cigarette	0.0564	0.3015	4 - 0
12	Falk5	2022	0 2 4 5 6	Never Use	Cigarette	0.7486	0.0779	5 - 0
12	Falk5	2022	0 2 4 5 6	Never Use	Dual	0.8738	0.0894	6 - 0
12	Falk5	2022	0 2 4 5 6	Former Cigarette	E-cigarette	-0.1959	0.3068	4 - 2
12	Falk5	2022	0 2 4 5 6	Former Cigarette	Cigarette	0.4963	0.0962	5 - 2
12	Falk5	2022	0 2 4 5 6	Former Cigarette	Dual	0.6215	0.1057	6 - 2
12	Falk5	2022	0 2 4 5 6	E-cigarette	Cigarette	0.6922	0.3114	5 - 4

12	Falk5	2022	0 2 4 5 6	E-cigarette	Dual	0.8174	0.3145	6 - 4
12	Falk5	2022	0 2 4 5 6	Cigarette	Dual	0.1252	0.1186	6 - 5
15	Patel1	2022	4 5 6	E-cigarette	Cigarette	-0.1398	0.0022	5 - 4
15	Patel1	2022	4 5 6	E-cigarette	Dual	-0.0087	0.0031	6 - 4
15	Patel1	2022	4 5 6	Cigarette	Dual	0.1310	0.0022	6 - 5
17	Hirschtick4	2023	0 4 5 6	Never Use	E-cigarette	0.5519	0.5836	4 - 0
17	Hirschtick4	2023	0 4 5 6	Never Use	Cigarette	0.8120	0.2050	5 - 0
17	Hirschtick4	2023	0 4 5 6	Never Use	Dual	0.1130	0.6204	6 - 0
17	Hirschtick4	2023	0 4 5 6	E-cigarette	Cigarette	0.2601	0.6186	5 - 4
17	Hirschtick4	2023	0 4 5 6	E-cigarette	Dual	-0.4389	0.8517	6 - 4
17	Hirschtick4	2023	0 4 5 6	Cigarette	Dual	-0.6990	0.6534	6 - 5

Supplementary Figure 1. Network map of adjusted effect pooling for myocardial infarction outcome.

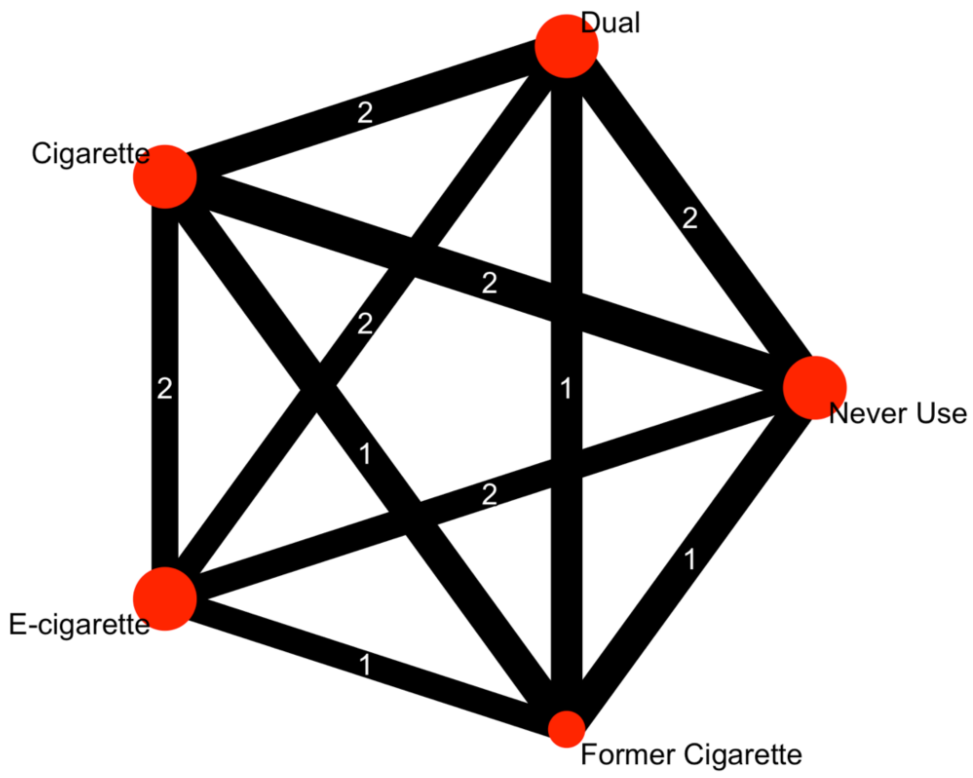

The size of the nodes is proportional to the number of included studies, while the thickness of the edges corresponds to the inverse standard errors of effect sizes comparing two exposures. The numbers within the edges indicate the number of studies for each comparison.

Supplementary Figure 2. Network map of adjusted effect pooling for stroke outcome.

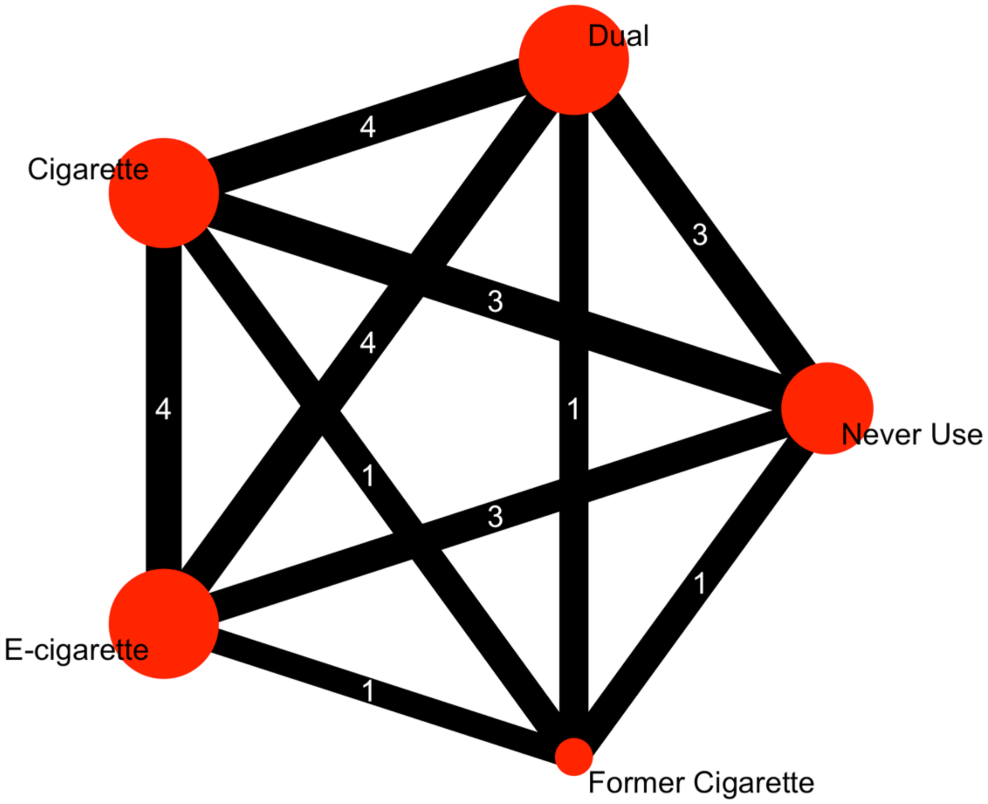

The size of the nodes is proportional to the number of included studies, while the thickness of the edges corresponds to the inverse standard errors of effect sizes comparing two exposures. The numbers within the edges indicate the number of studies for each comparison.

Supplementary Figure 3. Comparison-adjusted funnel plots of network meta-analysis of adjusted odds ratio for composite cardiovascular outcome

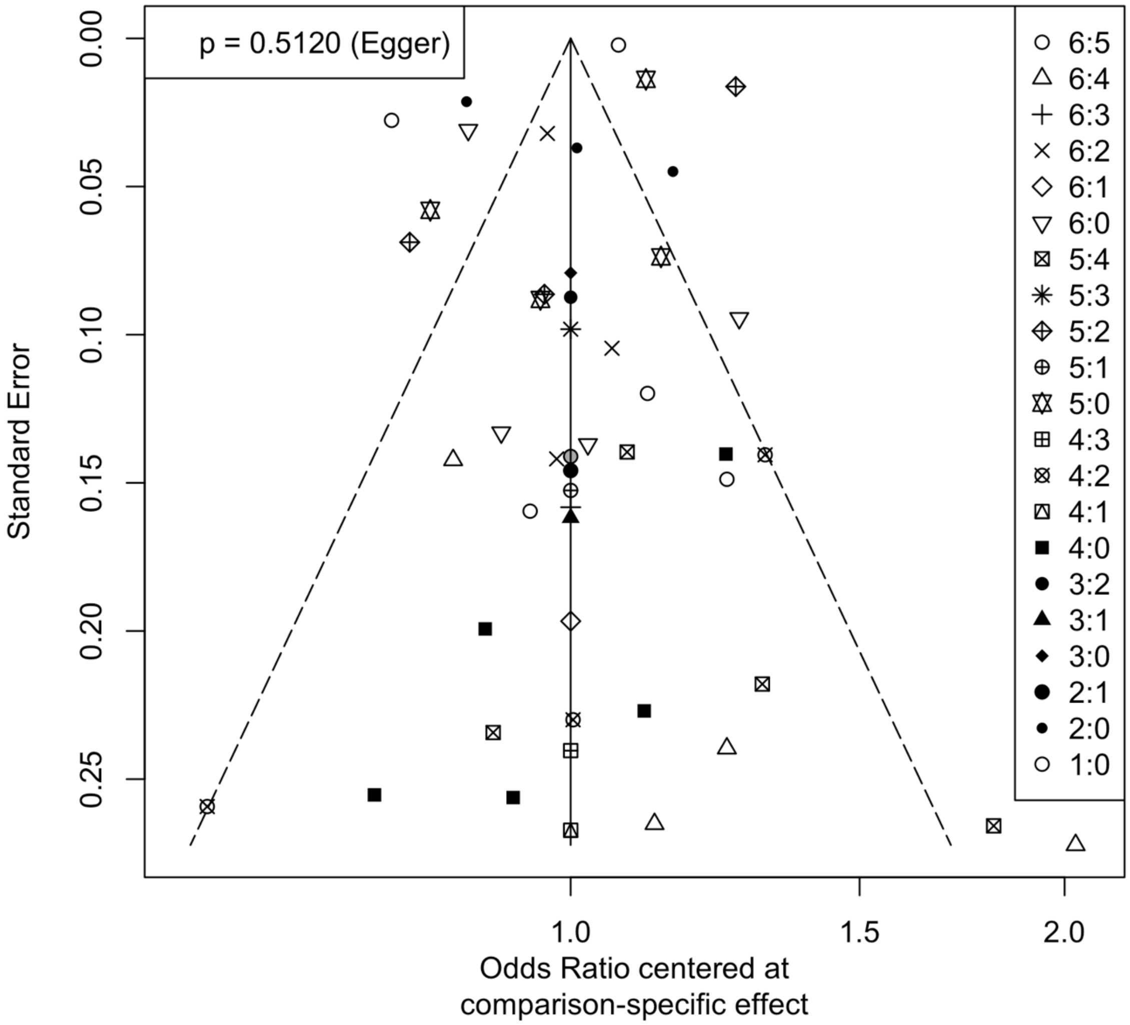

0 = Never Use, 1 = Former E-cigarette Use, 2 = Former Cigarette Use, 3 = Former Dual Use, 4 = E-cigarette Use, 5 = Cigarette Use, and 6 = Dual Use

Supplementary Figure 4. Comparison-adjusted funnel plots of network meta-analysis of adjusted odds ratio for myocardial infarction

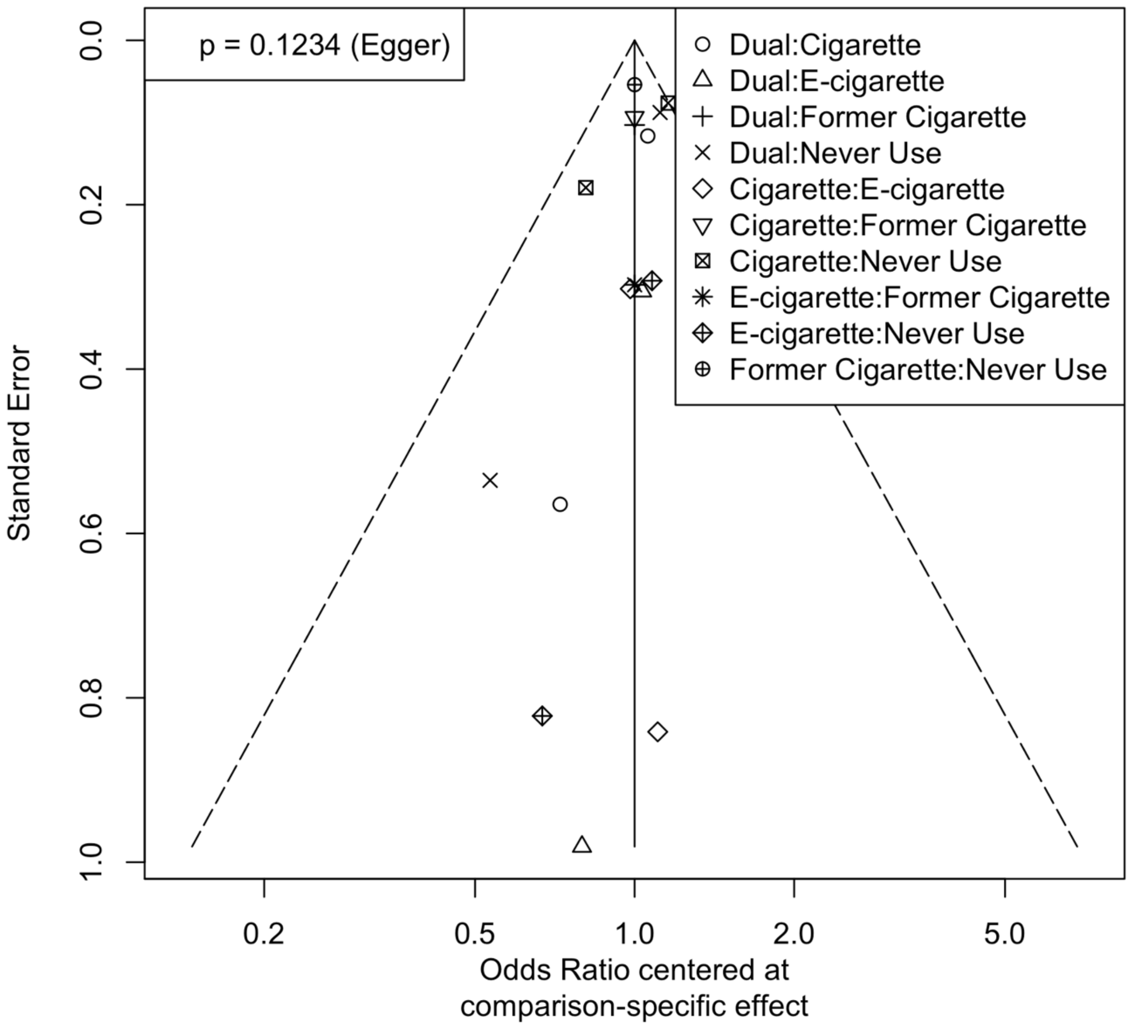

Supplementary Figure 5. Comparison-adjusted funnel plots of network meta-analysis of adjusted odds ratio for stroke

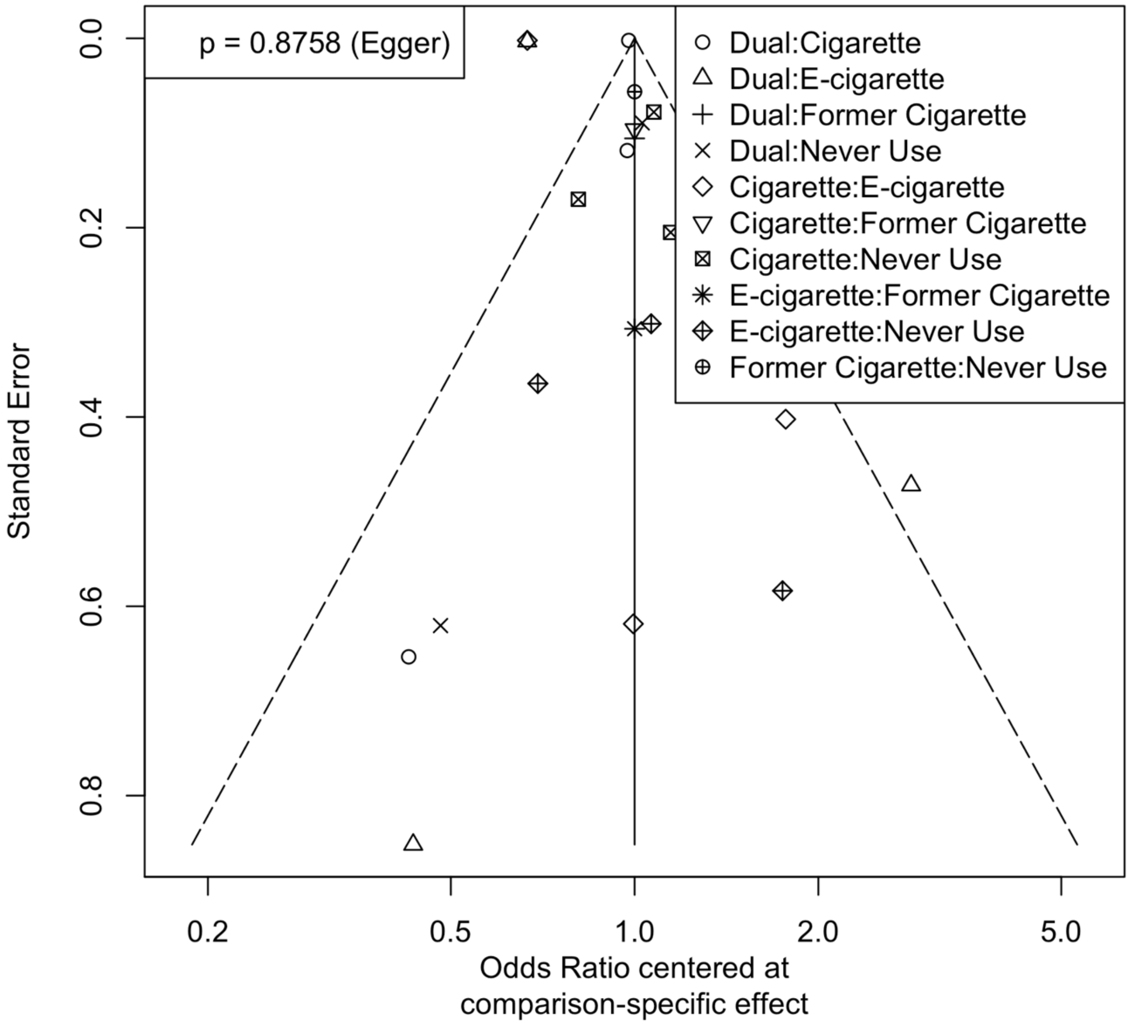

© 2025 Tansawet A. et al.
